# Molecular Characterization and Antibacterial Potential of Endophytic Fungal Isolates from Selected Mangroves along the Coastline of Kenya

**DOI:** 10.1155/2024/1261721

**Published:** 2024-09-06

**Authors:** Teresia Nyambura Wacira, Huxley Mae Makonde, Carren Moraa Bosire, Cromwell Mwiti Kibiti

**Affiliations:** ^1^ Department of Pure and Applied Sciences Technical University of Mombasa P.O. Box 90420-80100, Mombasa, Kenya; ^2^ Kenya Marine and Fisheries Research Institute P.O. Box 1881-40100, Kisumu, Kenya

## Abstract

The increasing emergence and re-emergence of resistant pathogenic microbes causes a health threat to the human population. Scientists have been striving to find novel bioactive compounds and drugs to overcome these obstacles. This study aimed to characterize mangrove endophytic fungi and evaluate their antibacterial activity. *Heritiera littoralis*, *Rhizophora mucronata, Bruguiera gymnorrhiza*, *Avicennia marina,* and *Xylocarpus granatum* species were collected from Tudor Creek, Mida Creek, and Gazi Bay. A total of 30 fungal isolates were subjected to molecular identification based on analysis of their ITS gene region. The isolates in the inferred phylogenetic trees were affiliated with the genus *Aspergillus*. Ethyl acetate and butanol crude extracts of 38.2% of the 76 isolated fungal endophytes and eight mycelia samples were screened for antibacterial activity against *Staphylococcus aureus* (ATCC 27853), *Escherichia coli* (ATCC 25922), and *Pseudomonas aeruginosa (*ATCC 25923) using the disc diffusion method. *A. marina* and *R. mucronata* harbored the most fungal endophytes that showed the highest antibacterial activity. Seven fungal broth extracts exhibited higher antibacterial activities against the tested microorganisms than the positive control. The minimum inhibitory concentration (MIC) activity for the isolates demonstrated that the ethyl acetate extract of a root endophytic fungal isolate (RC6) (3.31 ± 0.01) of *A. marina* is a strong inhibitor since it showed significantly lower MIC activity compared to the positive control (3.84 ± 0.00) against *Pseudomonas aeruginosa* (*P* < 0.05). Therefore, this study confirms that mangrove species harbor fungal isolates that have antibacterial activity and hence could serve as a novel source of antibiotics. It is recommended that the pure compounds from these extracts be isolated for further bioactivity tests and structural elucidation for consideration as lead molecules in drug discovery. In addition, the genes responsible for the enhanced bioactivity in these isolates can be characterized and bioengineered for pharmaceutical application.

## 1. Introduction

Throughout thousands of years and ages, nature has been a source of medicinal agents [1]. An impressive number of drugs in the modern world have been isolated from natural sources based on their use in traditional medicine [2]. Natural products remain a consistent source of drug and drug leads with several exciting molecules being reported from naturally occurring and genetically engineered microorganisms [3].

A recent survey conducted by China-based research groups reported that there has been an increase in the number of scientific publications on a large proportion of discovered marine bioactive compounds of microbial origin rather than plant origin [4]. The main featured microorganisms are endophytes from mangroves, marine algae, and marine invertebrates, making up more than 60% of the investigated microbial strains [4]. Out of 897 publications from 2009 to 2018, 80% strongly focused on fungi as a potential natural source of marine bioactive compounds [4]. Marine environment has been a center of focus lately since the oceans cover more than 70% of the world's surface and more than 15,000 unique natural compounds have been discovered from marine organisms [5]. It is predicted that there are over two million marine species, but only between 200,000 and 230,000 have been described [6]. The marine invertebrates make up over 75% of the described marine species and over 95% of all marine animal species [7]. This indicates that oceans offer unlimited potential for natural products and chemical diversity. Second, it provides quantitative habitat diversity, indicating that large populations of useful microorganisms are found in the ocean.

Furthermore, the majority of anticancer and antimicrobial drugs currently in clinical use or in development phases are natural molecules or their derivatives [8]. In comparison with other natural sources such as higher plants, microorganisms have not been greatly explored for human benefit [9].

Fungal endophytes are potential sources of bioactive compounds for medicinal purposes and there is a need to explore their diversity [10]. Fungal endophytes are important to plant growth by producing enzymes and secondary metabolites, which assist in the adaptation of plants to biotic stresses such as insects, herbivores, or invading pathogens and abiotic stresses such as drought and light [11]. Plants that grow in great biodiversity also have the potential for housing endophytes with great diversity and many endophytic fungi have been found to colonize the mangrove species [12]. Mangroves in special environments should be frequently studied for screening for the presence of endophytes that produce antimicrobial agents [13].

Some of the challenges to the development of therapeutics against diseases are resistance to the already existing antibiotics and hence less toxic and more potent antibiotics are required [14]. It is emphasized by modern researchers that there is a need to search for unexplored habitats such as forests [15], marine sponges [16], and mangroves [17] for pharmacologically active compounds. Mangrove endophytic fungi constitute the second largest group of marine endophytes [12] with leaves harboring a more diverse fungal endophytes community compared to other parts of the plant [18]. Endophytic fungi have been reported to be abundant in all tissues such as flowers, fruits, stems, roots, and leaves that are potential natural sources of bioactive compounds [19]. This study aimed at molecular characterization and determination of the antibacterial activity of endophytic fungi associated with selected mangrove plants on the Kenyan coast.

## 2. Materials and Methods

### 2.1. Sample Collection, Preparation, and Isolation of Mangrove Endophytic Fungi

Healthy leaves, aerial branches, and submerged roots of *Heritiera littoralis*, *Rhizophora mucronata, Bruguiera gymnorrhiza*, *Avicennia marina,* and *Xylocarpus granatum* mangrove species were collected from Mida Creek, Tudor Creek, and Gazi Bay along the Kenyan coastline. Isolation of the mangrove fungal endophytic was performed at the Kenya Marine and Fisheries Research Institute (KMFRI), Mombasa. Cultivation of the endophytic fungi was performed using potato dextrose agar (PDA) and the pH was adjusted to 4.8 by augmenting the medium with lactic acid to inhibit the bacterial growth, ensuring a selective environment for fungal proliferation. Subculturing each distinct colony further was performed to obtain pure cultures, which was crucial for the reliability of subsequent analyses [20].

A total of 30 fungal isolates out of 76 fungal isolates [20] were randomly selected based on their morphological characters and identified based on the analysis of the ITS gene region. In addition, twenty-nine (38.2%) of the fungal isolates were selected for their antibacterial activity testing considering the sample site, mangrove species, rate of growth (sporulation) in Potato Dextrose Broth (PDB), and the tissue part of the mangrove plant.

### 2.2. Molecular Characterization

#### 2.2.1. Genomic DNA Extraction

Approximately 100 mg of fungal mycelia from 5-day-old fungal pure cultures grown on PDA at room temperature was scraped out from the culture plates using a sterile surgical blade. A Quick-DNA bacterial/fungal kit (Zymo Research Corp. CA, United States) was used to extract the genomic DNA (gDNA) as described by the manufacturer, and the presence of the extracted gDNA was confirmed by running gel electrophoresis. In brief, a 1% agarose gel was prepared by weighing 1 gram agarose and mixing it into 100 ml of 1 × TAE buffer. The mixture was then heated to boil in a microwave and cooled down to 50°C. Samples were loaded in the gel and electrophoresis was conducted in 1 × TAE buffer at 90 units of voltage for 1 hour and visualized under ultraviolet after staining with ethidium bromide. To evaluate DNA purity, absorbance was observed from 230 nm to 320 nm to detect other possible contaminants (Eppendorf BioSpectrometer, Germany). The gDNA was stored at −20°C for further use.

#### 2.2.2. Polymerase Chain Reaction (PCR) Amplification of the ITS Gene and Sequencing

The PCR amplification of the fungal internal transcribed spacer (ITS) rDNA gene region from the gDNA was performed using ITS1 (5′TCCGTAGGTGAACCTTGCGG3′) and ITS4 (5′TCCTCCGCTTATTGATATGC 3′) primers [21]. Amplification proceeded in a 30-cycle PCR using the HotStarTaq Plus Master Mix Kit (QIAGEN, USA) with initial heating at 94°C for 3 minutes, followed by 30 cycles of denaturation at 94°C for 30 seconds, annealing at 53°C for 40 seconds, and extension at 72°C for 1 minute. The final elongation step was performed at 72°C for 5 minutes. The presence of PCR products was confirmed using 1% agarose gel electrophoresis in 1 × TAE buffer at 90 voltage for 1 hour and visualized under ultraviolet upon staining with ethidium bromide. The PCR products were purified using the QIAquick PCR Purification Kit protocol (QIAGEN, Germany) based on the manufacturer's instructions. The purified PCR products were stored at −20°C before being shipped for sequencing. Sequencing of the purified PCR products was performed using a commercial service provider (Inqaba Biotech, Pretoria, South Africa).

#### 2.2.3. Phylogenetic Analysis

The DNA sequences were trimmed and edited to obtain complete sequences using BioEdit software. A search for similar sequences using BLASTN [22] was performed at the National Center for Biotechnology Information (NCBI) GenBank: https://www.ncbi.nlm.nih.gov/nucleotide/ [23]. From the GenBank sequence database, the closest nucleotide sequences were retrieved and put onto a FASTA file format that had the other newly obtained sequences from the study. Subsequently, all the sequences were aligned using the Clustal Omega program (http://www.clustal.org) [24] against the nearest neighbors. A neighbor-joining tree of the aligned sequences was constructed [25] using MEGA X software [26]. Evolutionary distances were computed using the maximum composite Likelihood method [27]. To obtain statistical support values for the branches, bootstrapping [28] was conducted with 1000 replicates. All sites, including gaps in the sequence alignment, were excluded pairwise in the phylogenetic analysis. Using the resultant neighbor-joining tree, each isolate was assigned to the proper taxonomic group. The taxonomic assignment was confirmed at a 90% confidence level using the naïve Bayesian rRNA classifier on the RDP website [29].

#### 2.2.4. Extraction of Crude Extracts for Antibacterial Activity Screening

Plugs of agar with mycelia growth covering the surface of the inoculated PDA (HiMedia, Mumbai, India) (39 g in 1 L distilled water) were transferred aseptically into 500 ml Erlenmeyer flasks containing 100 ml of potato dextrose broth (TM Media, Rajasthan, India) (24 g in 1 L sterile seawater). The inoculated flasks were then incubated at 26°C and intermittently shaken at 200 rpm for 15 days. Cultures were harvested by filtering off the mycelium using a filter funnel and a Whatman # 1 filter paper.

The filtrate was extracted with 200 ml of ethyl acetate (EtOAc) and centrifuged at 8000 rpm for 10 minutes at room temperature, and the upper layer (ethyl acetate) was collected and poured into a sterile conical flask. The extraction was repeated three times with equal volumes of EtOAc. The filtrate was also fractionated with 200 ml of butanol and repeated thrice. The harvested mycelia cultures were frozen at −40°C overnight, freeze-dried in a freeze dryer (ULVAC Technologies, Methuen, Japan), and the powdered material (10 g) was extracted twice with ethyl acetate and butanol [30]. The ethyl acetate and butanol extracts for both fungal broth and mycelia were then evaporated in a vacuum using a rotary evaporator (BIOBASE Company, Jiangsu, China) and dried to form an oil residue. The crude extracts were then dissolved in 1 ml dimethyl sulphoxide (1% DMSO) for antibacterial bioassay and stored at −20°C.

#### 2.2.5. Screening for Antibacterial Activity

The crude extracts of the broth and fungal mycelia were screened for their antibacterial activity based on the Kirby–Bauer test [31]. The EtOAc and butanol extracts were tested against two Gram-negative bacteria: *Escherichia coli* (ATCC 25922) and *Staphylococcus aureus* (ATCC 27853) and one Gram-positive bacteria *Pseudomonas aeruginosa* (ATCC 25923) using the paper disk method and each treatment comprised of three replicates. The test microorganisms were sourced from the Kenya Medical Research Institute (KEMRI), Nairobi, Kenya. The cell suspensions of the bacteria were adjusted to approximately 10^5^ CFU/ml using McFarland standards.

A sterile cotton swab was dipped into the bacterial suspension. To remove excess liquid, the swab was rotated several times with firm pressure on the inside wall of the tube above the fluid levels. Using the swab, the Mueller–Hinton agar plate (MHA) (HiMedia, Mumbai, India) was streaked to form a bacterial lawn. To obtain uniform growth, the plate was streaked with the swab in one direction, rotated at 90°, and streaked again in that direction. The plate was allowed to dry for approximately 5 minutes.

Each of the 6 mm sterile Whatman antibiotic assay discs (Sigma-Aldrich Company, St. Louis, Germany) was impregnated with 2 *μ*l of crude extracts, negative control (DMSO), or positive control (streptomycin antibiotic). In each of the test plates, 20 mg/ml in distilled water of positive control (streptomycin antibiotic), negative control (DMSO), ethyl acetate, and butanol crude extracts (10 mg/ml in DMSO) was placed in triplicate. The discs were placed on the surface of the medium containing 10^5^ cells of bacteria test strains and the plates were kept in the biosafety cabinet for 5 minutes to allow the agents to diffuse into the agar. After overnight incubation at 37°C, the diameter of zones of inhibition was measured in millimeters using a ruler.

### 2.3. Minimum Inhibitory Concentration (MIC)

Seven endophytic fungal extracts that displayed the most promising antimicrobial activity against the test organisms (*E. coli, S. aureus,* and *P. aeruginosa*) compared to the positive control were selected for MIC analysis using the broth dilution method described in [32]. Five tubes were used; to each tube, 2 ml of the sterile nutrient broth (NB) was placed in the 2^nd^ to 5^th^ tubes (except in tube 1). To tubes 1 and 2, 2 ml of the endophytic fungal extracts (10 mg/ml) was placed and a 2-fold serial dilution was performed from the 2^nd^ to 5^th^ tubes, while 2 ml was discarded from the 5^th^ tube to obtain 0.625 mg/ml, 1.25 mg/ml, 2.5 mg/ml, 5 mg/ml, and 10 mg/ml concentrations. An equal volume of 0.2 ml broth culture of 0.5 McFarland turbid-identified bacteria was added to all the tube dilution ranges. An overnight broth culture of 0.2 ml was placed in a positive control tube and 2 ml of NB was added to serve as a control for testing the growing ability of the medium. 2 ml nutrient broth was added to serve as a negative control for testing the sterility of the medium and the equipment.

The turbidity of each tube (optical density) was measured at zero hour (*T*_0_) using a UV-VIS spectrophotometer (Konik, Barcelona, Spain) at 620 nm wavelength. The tubes were incubated at 37°C for 24 hours and the absorbance of each tube which is proportional to the turbidity of the bacterial growth was noted [33]. The MIC was defined and recorded as the lowest concentration of the test antibacterial agent that had inhibition on 50% bacterial growth. This was determined by assessing growth by calculating the difference in absorbance between the test tubes and the control tubes that had the broth and antibacterial agent alone without the test bacteria.

### 2.4. Data Analysis

All the experiments were carried out in triplicate. Two-way ANOVA was performed to test whether or not there were significant differences in the diameter of zones of inhibition between the different extracts of fungal endophytes recovered from the mangrove species. The zones of inhibition were presented as mean ± standard deviation (SD).

Furthermore, statistical tests of means with the same superscripts (^∗^) within the column were considered not significantly different from one another (Fisher LSD at 95% confidence) after a post hoc analysis using Minitab software version 21.4.1. The differences were decided as significantly different with the condition *P* < 0.05 (*α* = 0.05).

## 3. Results

### 3.1. Affiliation of ITS Gene Sequences of the Isolates

**Figure 1:**
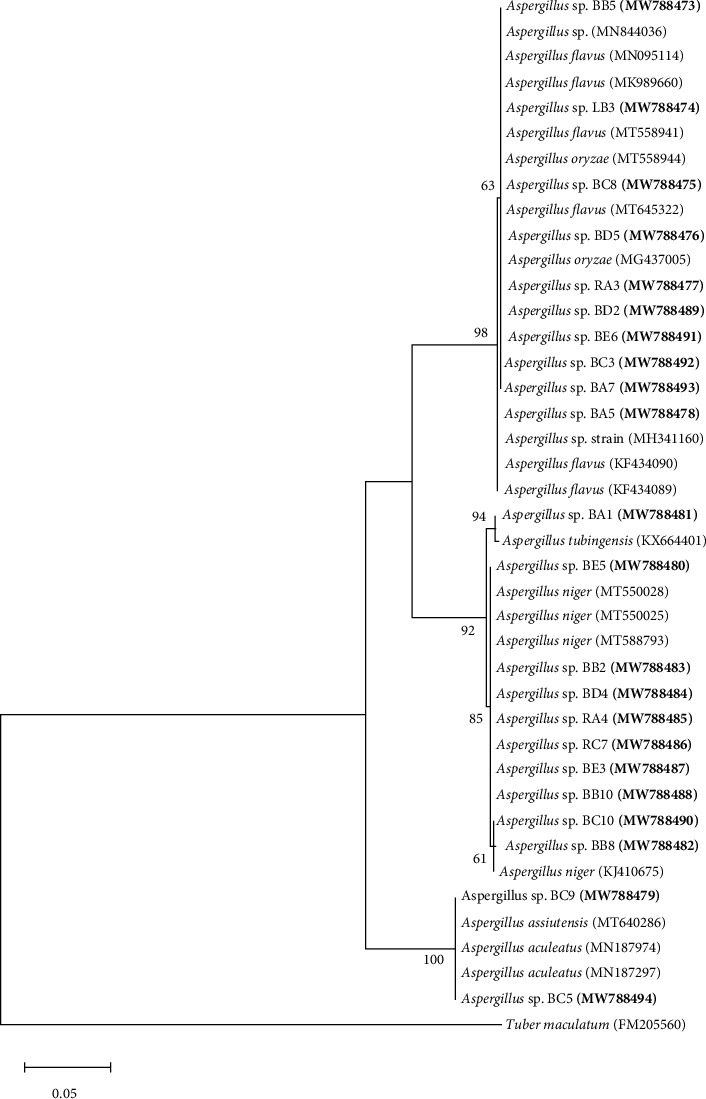
Evolutionary relationships between partial ITS gene sequences of the isolates and some closest known fungal species. *Tuber maculatum* (accession no. FM205560) was used to root the phylogenetic tree. Bootstrap values (>50%) based on 1000 replications are shown at branch nodes. Bar, 0.05 substitutions per nucleotide position.

A total of 30 endophytic isolates were analyzed. The isolates (with their accession numbers in parenthesis) in the inferred phylogenetic trees were affiliated with the genus *Aspergillus,* belonging to the fungal phylum *Ascomycota* (Figure 1). Comparison of the newly isolated ITS gene sequences with known sequences in the GenBank database using BLASTn analysis indicated sequences similarities of >98% with known sequences in the nucleotide sequence database (Table 1). All the isolates were affiliated with several known fungal species from the genus *Aspergillus* with >98% sequence identity (Table 1). Ten isolates (accession numbers MW788473 to MW788478, MW788489, and MW788491 to MW788493) had between 98 and 100% sequence identities with known *Aspergillus* species (*Aspergillus flavus* and *Aspergillus oryzae*) and together formed a single subcluster supported with a bootstrap value of 98% (Figure 1; Table 1). Out of the ten isolates, BB5 (MW788473) from Mida Creek and LB3 (MW788474) from Tudor Creek were recovered from a branch and leaf of *R. mucronata,* respectively. Isolates RA3 (MW788477) from Mida Creek and BA5 (MW788478) from Tudor Creek were recovered from a root and branch of *B. gymnorrhiza,* while isolates BD5 (MW788476) and BD2 (MW788489) were recovered from a branch of *X. granatum* from Gazi Bay. Isolates BE6 (MW788491), BC3 (MW788492), and BA7 (MW788493) were recovered from the branches of *H. littoralis, A. marina,* and *B. gymnorrhiza,* respectively (Table 1).

Nine isolates (BE5 (MW788480), BB8 (MW788482), BB2 (MW788483), BD4 (MW788484), RA4 (MW788485), RC7 (MW788486), BE3 (MW788487), BB10 (MW788488), and BC10 (MW788490)) had >98% sequence identities with *Aspergillus niger* (Table 1) and together formed a minor subcluster supported with a bootstrap value of 92% (Figure 1). Out of the nine, three isolates (BB8 (MW788482), BB2 (MW788483), and BB10 (MW788488)) were obtained from the branches of *R. mucronata.* Two isolates (RC7 (MW788486) and BC10 (MW788490)) were recovered from a root and branch of *A. marina,* while isolates BE5 (MW788480) and BE3 (MW788487) were recovered from the branches of *H. littoralis.* Isolates BD4 (MW788484) and RA4 (MW788485) were recovered from *X. granatum* and *B. gymnorrhiza,* respectively (Table 1). Within the same subcluster, isolate BA1 (MW788481), which was recovered from a branch of *B. gymnorrhiza,* formed a minor subcluster with *Aspergillus tubingensis* (KX664401) (Figure 1).

Two isolates, namely, BC9 (MW788479) and BC5 (MW788479) were closely related to *Aspergillus assiutensis* with 99% sequence identity and together formed a separate subcluster supported with a bootstrap value of 100% (Figure 1). These isolates (BC9 and BC5) were both obtained from the branches of *A. marina* from Mida Creek (Table 1).

### 3.2. Screening for Antibacterial Activity

The fungal broth and mycelia were tested against three human pathogenic strains of Gram-positive bacteria *S. aureus* (ATCC 27853) and Gram-negative bacteria *Escherichia coli* (ATCC 25922) and *P. aeruginosa* (ATCC 25923) (Tables 2, 3, and 4).

Seven fungal broth crude extracts (LB4, BD4, BE5, BA11, RC6, RC3, and LB1) out of the twenty-nine exhibited high antibacterial activity against the three tested microorganisms and were more effective than the positive control (Tables 2 and 3).

The ethyl acetate extracts of the endophytic fungal isolates LB4, BD4, BE5, and BA11 exhibited high antibacterial activities against all test microorganisms compared to the positive control (Table 2). The extracts of RC6 (29.17 ± 0.1 mm) and RC3 (29.17 ± 0.1 mm) showed a wide variety of antibacterial activity against *P. aeruginosa* compared to the positive control.

The ethyl acetate extracts of LB4 (41.03 ± 0.1), BD4 (39.07 ± 0.1), BE5 (34.03 ± 0.1), and BA11 (36.03 ± 0.1) showed a comparable higher antibacterial activity against *E. coli* than the positive control (Table 2). The inhibitory activity of the ethyl acetate extracts against *P. aeruginosa* was observed as LB4 (35.07 ± 0.1 mm), BD4 (35.07 ± 0.1 mm), BE5 (32.13 ± 0.1 mm) and BA11 (39.03 ± 0.1 mm) compared to the positive control.

A wide range of antibacterial activity was exhibited by ethyl acetate extracts of LB4 (44.07 ± 0.1 mm), BD4 (31.07 ± 0.1 mm), BE5 (35.07 ± 0.1 mm), and BA11 (39.03 ± 0.1 mm) against *S. aureus* compared to the positive control.

Butanol extracts of isolate LB1 (29.07 ± 0.1 mm) showed a wide range of antibacterial activities against *P. aeruginosa* compared to the positive control (28.30 ± 0.2 mm) (Table 3).

In the fungal mycelia, ethyl acetate extract of sample M2 was more effective in *S. aureus* compared to other extracts (Table 4). Butanol extract of sample M8 was effective in *S. aureus* and *E. coli* but not in *P. aeruginosa*.

### 3.3. Minimum Inhibitory Concentration (MIC)

The varying concentrations between 0.625 mg/ml and 10 mg/ml of the endophytic fungal extracts were tested to determine their MICs (Table 5). The lowest MIC (2.86 ± 0.01) in the ethyl acetate extract of isolate BE5 was observed against *E. coli*. The butanol extract of isolate LB1 showed the least MIC activity (4.57 ± 0.01) against *P. aeruginosa*. The ethyl acetate extracts of isolates BE5, BA11, LB4, and RC6 showed significantly lower MIC activity (Table 5) compared to the standard drug/control (streptomycin) against test microorganisms (*p* < 0.05).

The ethyl acetate extracts of isolate BE5 had a MIC of 2.86 ± 0.01 compared to the control (3.77 ± 0.00) against *E. coli.* The MIC (3.05 ± 0.01) for the ethyl acetate extracts of isolate BA11 against *S. aureus* was lower than the positive control (4.5 ± 0.00). For the isolate LB4, the minimum inhibitory concentrations of 3.23 ± 0.01 and 3.69 ± 0.01 were observed against *P. aeruginosa* and *S. aureus,* respectively, compared to the positive controls that had minimum inhibitory concentrations of 3.84 ± 0.00 and 4.5 ± 0.00, respectively. A lower MIC (3.31 ± 0.01) for isolate RC6 was observed compared to the positive control (3.84 ± 0.00) against *P. aeruginosa.*

The MIC activity was classified as strong inhibitors (MIC up to 0.5 mg·ml^−1^), moderate inhibitors (MIC between 0.6 and 1.5 mg·ml^−1^), and weak inhibitors (MIC above 1.6 mg·ml^−1^).

The root endophytic fungal isolate ethyl extract of *A. marina* was a strong inhibitor of *P. aeruginosa*. The moderate inhibitors were ethyl extract of leaf endophytic fungi isolate of *R. mucronata* against *P. aeruginosa* and *S. aureus*, followed by branch isolate extract of *H. littoralis* against *E. coli*, extract of branch isolates of *B. gymnorrhiza* against *S. aureus*, and branch isolate extract of *H. littoralis* against *S. aureus*.

The butanol extract against *P. aeruginosa* of the leaf endophytic fungi isolates of *R. mucronata* against *P. aeruginosa* and the branch isolate of *B. gymnorrhiza* were weak inhibitors in the MIC activity. The ethyl acetate extract's weak inhibitor was the branch endophytic fungi isolate of *X. granatum* against all test microorganisms (*E. coli*, *P. aeruginosa,* and *S. aureus*) and the root isolate of *A. marina*.

## 4. Discussion

The study characterized mangrove endophytic fungi and evaluated their antibacterial activity. It has been asserted that plant species could serve as a dependable reservoir of novel endophytes containing abundant antimicrobial metabolites [42]. This study confirmed that *B. gymnorrhiza, H. littoralis, A. marina, R. mucronata,* and *X. granatum* mangrove species harbor fungal isolates that have antibacterial activity and hence could serve as a novel source of antibiotics. The findings are consistent with those of previous studies that documented the antimicrobial activities of plant endophytic fungi and actinomycetes [43, 44], respectively. In addition, the authors in [45] recognized endophytic fungi as valuable reservoirs of diverse bioactive secondary metabolites possessing antimicrobial properties.

A total of 30 fungal isolates were randomly selected as morphotypes of the genus *Aspergillus* according to [46] since they were the most dominant isolates in all the five mangrove species.

This study revealed that *Aspergillus niger* is the predominant fungal species associated with the *B. gymnorrhiza, H. littoralis, A. marina, R. mucronata,* and *X. granatum* mangrove species. A similar study along the coast of South Andaman Sea, Andaman and Nicobar Islands, India reported that *A. niger* was prominent with a trend of rapid growth [47]. Other *Aspergillus* species reported in this study were *A. flavus, A. oryzae, A. aculeatus, A. tubingensis, A. assiutensis,* and several unidentified *Aspergillus* spp.

The phylogenetic analysis revealed three main clusters that were represented by the following sections of aspergilli: *A. niger, A. flavus, A. oryzae, A. aculeatus, A. tubingensis,* and *A. assiutensis*. One cluster comprised of *A. oryzae* and *A. flavus* confirmed the evolutionary closeness between the two species. The close genetic relatedness between *A. oryzae* and *A. flavus* was also recorded by [48]. A study on the diversity of fungi from mangroves of the Mahanadi delta, Orissa, India, found *A. oryzae, A. flavus,* and *A. niger* to be common in the area [49]. Another main cluster consisted of the filamentous black aspergilli in the Nigri section. Within this cluster was a minor subcluster, which comprised *A. niger*, *A. tubingensis,* and *A.* sp. BA1 (MW788481). *A. niger* and *A. tubingensis* are both classified as biseriate species [50]. *A. niger* was found in abundance compared to the other species recorded in this study. The last main cluster consisted of *A. assiutensis* and *A. aculeatus*. *A. aculeatus* and *A. niger* were previously found in the soil of grapevine plantations [51]. *A. assiutensis* was reported in ElKhawaled village, Sahel-Saleem city from the air of grapevine plantation by [52].

The results on antibacterial activity showed that ethyl acetate was the most effective solvent compared to butanol. The endophytic fungal extracts of isolates LB4, BD4, BE5, BA11, RC6, RC3, and LB1 exhibited high antibacterial activities against the test microorganisms compared to the positive control. In this study, *P. aeruginosa* exhibited the highest susceptibility among the tested bacterial strains. It was particularly sensitive to all the seven endophytic fungal extracts that surpassed the antibacterial activity compared to the positive control. This finding suggests that these mangrove fungal isolate extracts hold promise as potential antimicrobial agents against *P. aeruginosa* infections. Similarly, another study in Kenya reported that extracts from fungal isolates of four mangrove species found in Mida Creek and Gazi Bay showed antibacterial activity against the pathogenic bacterial strains including *P. aeruginosa* [53].

Among the five mangrove species sampled, root isolates of *A.* marina (RC6 and RC3) and leaf isolates of R. mucronata (LB4 and LB1) had the most effective extracts against the test microorganisms compared to the positive control. This demonstrates that the mangrove endophytes are potential reservoirs of bioactive compounds, which can be lead molecules for drug discovery. Elsewhere, the authors in [54] found that 34 ethyl acetate crude extracts from 70 strains of mangrove-associated fungi, isolated from the mangrove plant (*Laguncularia racemose*) exhibited activity against a range of pathogenic bacteria. Moreover, the antibacterial activity of leaf extract of mangroves including *R. mucronata* from Chorao Island, Goa, was investigated against human bacterial pathogens and it was reported to be a potential source for the development of novel antibiotics [55]. Ethyl acetate root extracts of *A.* marina have also been found in the Kingdom of Saudi Arabia and observed to have antibacterial activities against *E. coli, S. aureus,* and *P. aeruginosa* [56]. All these results underpin that mangrove species harbor fungal isolates that are good sources of bioactive compounds.

From this study, ethyl acetate and butanol broth extracts isolated from leaves found in Mida Creek were the most effective against S. *aureus* and *P. aeruginosa,* respectively, compared to all the other isolates. Microplastic pollution poses a threat to the mangrove blue carbon ecosystem at Mida Creek affecting not only the water quality but also the organisms that reside within the habitat [57]. According to a recent study, microplastic pollution is not only threatening the mangroves but also the microbial communities that reside within them. As a result, these microorganisms experience significant changes and undergo substantial changes when exposed to microplastics [58]. Fungal extracts isolated from the mangrove leaves in Luzon Island, Philippines, showed antibacterial activity against the Gram-positive bacteria *S. aureus* [59]. In our study, both ethyl acetate and butanol mycelial extracts were most effective against *S. aureus.* Synytsya [31] observed that extracts from the fungal mycelia had properties.

The ethyl acetate extracts of isolates BE5, BA11, LB4, and RC6 showed a significantly lower MIC activity compared to the standard drug/control (streptomycin) against test microorganisms. Therefore, branch isolates of *X. granatum* and *B. gymnorrhiza* species, leaf isolates of *R. mucronata* species, and root isolates of *A. marina* species had more inhibitory effects on the tested microorganisms.

The phylum *Ascomycota* appears to be the predominant group among endophytic fungi communities when isolated using standard protocols [60, 61], a finding consistent with our study. It has been noted that fungi from certain other phyla may be influenced by the culturing method [60]. Comparative studies have indicated that only a fraction of microorganisms in nature are amenable to cultivation using traditional microbiological techniques [62]. Various factors, including insufficient knowledge of their nutritional requirements and the demanding nature of microorganisms, particularly those from environmental sources, can hinder their growth in laboratory conditions [63]. We anticipate that the culturing methods used in the current study to isolate the fungal endophytes might have been influenced by these factors. Although novel molecules have been isolated, marine-derived fungi, including those associated with mangroves, continue to be underrepresented as sources of new natural products [64]. Therefore, further screening of these microbes, particularly mangrove fungal endophytes, for biological activities is highly recommended.

## 5. Conclusion

From this study, it is concluded that *Aspergillus niger* was the predominant fungal endophyte associated with the *R. mucronata*, *B. gymnorrhiza*, *A. marina*, *X. granatum*, and *H. littoralis* mangrove species. It is also evident that genus *Aspergillus* has a high adaptability to various ecological environments and cytotoxic studies to determine if the extracts are safe and would be useful. Some fungal isolates have antibacterial activity and hence results generated form the basis of future efforts on drug discovery.

## Figures and Tables

**Table 1:** Affiliation of endophytic fungi with their closest taxonomic relatives and their associated host mangrove species.

Sample ID	Accession no.	Host mangrove species	Location	Closest taxonomic affiliation	Isolation source	Country	% ID
BB5	MW788473	R. mucronata	Mida Creek	*Aspergillus* sp. (MN844036)	Rubia plant endophyte [34]	China	99
LB3	MW788474	R. mucronata	Tudor Creek	*Aspergillus flavus* (MT558941)	Rosy rice vinegar solid mash [35]	India	100
BC8	MW788475	A. marina	Tudor Creek	*Aspergillus flavus* (MT645322)	Insect larva [36]	China	100
BD5	MW788476	*X. granatum*	Gazi Bay	*Aspergillus oryzae* (MG437005)	Coastal marine sediment [37]	India	100
RA3	MW788477	*B. gymnorrhiza*	Mida Creek	*Aspergillus flavus* (MT558941)	Rosy rice vinegar solid mash [35]	India	100
BA5	MW788478	*B. gymnorrhiza*	Tudor Creek	*Aspergillus* sp. (MH341160)	Animal leather [38]	Pakistan	99
BC9	MW788479	A. marina	Mida Creek	*Aspergillus aculeatus* (MN187974)	Marine estuary, Tamil Nadu [37]	India	99
BE5	MW788480	*H. littoralis*	Gazi Bay	*Aspergillus niger* (mk534501)	Leachate contaminated soil [39]	Malaysia	100
BA1	MW788481	*B. gymnorrhiza*	Mida Creek	*Aspergillus tubingensis* (KX664401)	Floor surface [40]	USA	99
BB8	MW788482	R. mucronata	Gazi Bay	*Aspergillus niger* (kj410675)	Soil from stevia plants [41]	India	100
BB2	MW788483	R. mucronata	Mida Creek	*Aspergillus niger* (mt620753)	Rice wine [35]	China	100
BD4	MW788484	*X. granatum*	Gazi Bay	*Aspergillus niger* (mt588793)	Plant [35]	India	100
RA4	MW788485	*B. gymnorrhiza*	Mida Creek	*Aspergillus niger* (mt620753)	Rice wine [35]	China	100
RC7	MW788486	A. marina	Mida Creek	*Aspergillus niger* (mt588793)	Plant [35]	India	100
BE3	MW788487	*H. littoralis*	Gazi Bay	*Aspergillus niger* (MK534501)	Leachate contaminated soil [39]	Malaysia	100
BB10	MW788488	R. mucronata	Tudor Creek	*Aspergillus niger* (mt550026)	N/A	Thailand	100
BD2	MW788489	*X. granatum*	Gazi Bay	*Aspergillus oryzae* (MT558944)	Zhejiang rosy rice vinegar [35]	China	100
BC10	MW788490	R. mucronata	Gazi Bay	*Aspergillus niger* (mt588793)	Soil from stevia plants [41]	India	99
BE6	MW788491	*H. littoralis*	Gazi Bay	*Aspergillus oryzae* (MT558944)	Zhejiang rosy rice vinegar [35]	China	100
BC3	MW788492	A. marina	Tudor Creek	*Aspergillus oryzae* (MT558944)	Zhejiang rosy rice vinegar [35]	China	100
BA7	MW788493	*B. gymnorrhiza*	Tudor Creek	*Aspergillus flavus* (MT584825)	N/A	China	100
BC5	MW788494	A. marina	Mida Creek	*Aspergillus aculeatus* (MN187297)	Marine estuary [37]	India	99

**Table 2:** Antibacterial activities of ethyl acetate extracts from mangrove endophytic fungi.

Sample ID	Endophytic fungal isolate	Site sampled	Host plant	Tissue type	Ethyl acetate extracts' inhibition zone diameters (mm)		
					Gram-negative bacteria		Gram-positive bacteria
					*Escherichia coli* (ATCC 25922)	*Pseudomonas aeruginosa* (ATCC 27853)	*Staphylococcus aureus* (ATCC 25923)
LA1	*Alternaria*	Mida Creek	*B. gymnorrhiza*	L	0.00 ± 0.0^f^	7.03 ± 0.1^d^	14.07 ± 0.1^abcd^
LB1	*Alternaria*	Mida Creek	*R. mucronata*	L	7.07 ± 0.1^ef^	8.17 ± 0.2^cd^	10.30 ± 0.1^bcd^
BB3	*Alternaria*	Tudor Creek	*R. mucronata*	B	19.03 ± 0.1^bcde^	19.17 ± 0.2^bcd^	11.03 ± 0.1^bcd^
BB6	*Alternaria*	Mida Creek	*R. mucronata*	B	0.00 ± 0.0^f^	0.00 ± 0.0^d^	0.00 ± 0.0^d^
	Negative control				0.00 ± 0.0^f^	0.00 ± 0.0^d^	0.00 ± 0.0^d^
	Positive control				28.10 ± 0.1^bcde^	28.30 ± 0.2^abc^	31.70 ± 0.1^abc^
LC3	*Penicillium*	Mida Creek	*A. marina*	L	0.00 ± 0.0^f^	8.03 ± 0.1^cd^	9.07 ± 0.1^bcd^
LC5	*Penicillium*	Tudor Creek	*A. marina*	L	10.13 ± 0.1^def^	0.00 ± 0.0^d^	14.07 ± 0.1^abcd^
RC6	*Penicillium*	Mida Creek	*A. marina*	R	14.10 ± 0.1^def^	29.17 ± 0.1^abc^	26.07 ± 0.1^abcd^
	Negative control				0.00 ± 0.0^f^	0.00 ± 0.0^d^	0.00 ± 0.0^d^
	Positive control				32.20 ± 0.1^abcd^	27.09 ± 0.1^abc^	37.10 ± 0.1^ab^
LB2	*Cladosporium*	Mida Creek	*R. mucronata*	L	20.03 ± 0.1^bcde^	7.03 ± 0.1^d^	0.00 ± 0.0^d^
LB4	*Cladosporium*	Tudor Creek	*R. mucronata*	L	41.03 ± 0.1^a^	35.07 ± 0.1^ab^	44.07 ± 0.1^a^
RA2	*Cladosporium*	Mida Creek	*B. gymnorrhiza*	R	0.00 ± 0.0^f^	10.03 ± 0.1^cd^	0.00 ± 0.0^d^
	Negative control				0.00 ± 0.0^f^	0.00 ± 0.0^d^	0.00 ± 0.0^d^
	Positive control				35.40 ± 0.1^abc^	28.07 ± 0.1^abc^	37.40 ± 0.1^ab^
RA3	*Aspergillus*	Mida Creek	*B. gymnorrhiza*	R	26.07 ± 0.1^bcde^	29.07 ± 0.1^abc^	22.10 ± 0.1^abcd^
BA5	*Aspergillus*	Tudor Creek	*B. gymnorrhiza*	B	9.03 ± 0.1^ef^	10.17 ± 0.1^cd^	14.07 ± 0.1^abcd^
BA6	*Aspergillus*	Mida Creek	*B. gymnorrhiza*	B	6.03 ± 0.1^ef^	10.67 ± 0.1^cd^	0.00 ± 0.0^d^
BB8	*Aspergillus*	Gazi Bay	*R. mucronata*	B	0.00 ± 0.0^f^	19.03 ± 0.1^bcd^	15.03 ± 0.1^abcd^
BC3	*Aspergillus*	Gazi Bay	*A. marina*	B	10.67 ± 0.1^def^	11.03 ± 0.1^cd^	8.07 ± 0.1^bcd^
BC8	*Aspergillus*	Tudor Creek	*A. marina*	B	14.10 ± 0.1^def^	8.07 ± 0.1 ^cd^	9.03 ± 0.1^bcd^
BC9	*Aspergillus*	Mida Creek	*A. marina*	B	10.03 ± 0.1^def^	10.03 ± 0.1 ^cd^	13.07 ± 0.1^bcd^
BD4	*Aspergillus*	Gazi Bay	*X. granatum*	B	39.07 ± 0.1^ab^	35.07 ± 0.1^ab^	31.07 ± 0.1^abc^
BE5	*Aspergillus*	Gazi Bay	*H. littoralis*	B	34.03 ± 0.1^abc^	32.13 ± 0.1^ab^	35.07 ± 0.1^abc^
	Negative control				0.00 ± 0.0^f^	0.00 ± 0.0^d^	0.00 ± 0.0^d^
	Positive control				28.30 ± 0.1^bcde^	29.30 ± 0.4^abc^	29.20 ± 0.3
BA10	*Fusarium*	Gazi Bay	*B. gymnorrhiza*	B	0.00 ± 0.0^f^	12.07 ± 0.1^cd^	10.07 ± 0.1^bcd^
BC6	*Fusarium*	Mida Creek	*A. marina*	B	0.00 ± 0.0^f^	0.00 ± 0.0^d^	11.03 ± 0.1^bcd^
	Negative control				0.00 ± 0.0^f^	0.00 ± 0.0^d^	0.00 ± 0.0^d^
	Positive control				21.03 ± 0.1^bcde^	24.10 ± 0.1^abc^	28.60 ± 0.1^abcd^
RB4	*Lasiodiplodia*	Mida Creek	*R. mucronata*	R	0.00 ± 0.0^f^	10.07 ± 0.1^cd^	15.07 ± 0.1^abcd^
BB4	*Lasiodiplodia*	Tudor Creek	*R. mucronata*	B	9.03 ± 0.1^ef^	8.03 ± 0.1^cd^	18.07 ± 0.1^abcd^
BB7	*Lasiodiplodia*	Mida Creek	*R. mucronata*	B	16.03 ± 0.1^def^	25.10 ± 0.1^abc^	21.13 ± 0.1^abcd^
	Negative control				0.00 ± 0.0^f^	0.00 ± 0.0^d^	0.00 ± 0.0^d^
	Positive control				25.70 ± 0.1^bcde^	30.10 ± 0.1^abc^	39.70 ± 0.1^a^
BA8	*Nigrospora*	Mida Creek	*B. gymnorrhiza*	B	0.00 ± 0.0^f^	9.03 ± 0.1^cd^	9.03 ± 0.1^bcd^
BA11	*Nigrospora*	Tudor Creek	*B. gymnorrhiza*	B	36.03 ± 0.1^abc^	39.03 ± 0.1^a^	39.03 ± 0.1^a^
BC7	*Nigrospora*	Mida Creek	*A. marina*	B	11.03 ± 0.1^def^	0.00 ± 0.0^d^	0.00 ± 0.0^d^
RC3	*Nigrospora*	Mida Creek	*A. marina*	R	26.07 ± 0.1^bcde^	29.17 ± 0.1^abc^	22.03 ± 0.1^abcd^
	Negative control				0.00 ± 0.0^f^	0.00 ± 0.0^d^	0.00 ± 0.0^d^
	Positive control				34.60 ± 0.1^abc^	27.00 ± 0.1^abc^	30.10 ± 0.1^abc^
BC2	*Chaetomium*	Gazi Bay	*A. marina*	B	10.07 ± 0.1^def^	19.03 ± 0.1^cd^	17.03 ± 0.1^abcd^
	Negative control	0.00 ± 0.0^f^	0.00 ± 0.0^d^	0.00 ± 0.0^d^			
	Positive control	25.03 ± 0.1^bcde^	25.03 ± 0.1^abc^	28.07 ± 0.1^abcd^			

Isolates are coded depending on the part of the tree sampled; prefix letters L, B, and R representing leaf, branch, and root tissue of the mangrove species, *respectively*, followed by letters A, B, C, D, and E representing *B. gymnorrhiza, R. mucronata, A. marina, X. granatum,* and *H. littoralis mangrove species, respectively,* and then a number of the sample collected from the mangrove species was isolated. Means followed by the same superscript letter(s) in each group are not significantly different at *α* = 0.05 (Fisher LSD, 95% confidence). The significance of each group is represented by superscript letters (a, b, and c). The distinct letters indicate statistically significant differences between the groups. Therefore, when two groups are assigned different superscript letters, their results are statistically different, and any observed difference between them is considered significant.

**Table 3:** Antibacterial activities of butanol extracts from mangrove endophytic fungi.

Sample ID	Endophytic fungal isolate	Site sampled	Host plant	Tissue type	Butanol extracts' inhibition zone diameters (mm)		
					Gram-negative bacteria		Gram-positive bacteria
					*Escherichia coli* (ATCC 25922)	*Pseudomonas aeruginosa* (ATCC 27853)	*Staphylococcus aureus* (ATCC 25923)
LA1	*Alternaria*	Mida Creek	*B. gymnorrhiza*	L	9.03 ± 0.1^ab^	10.13 ± 0.2^ab^	9.03 ± 0.1^bcd^
LB1	*Alternaria*	Mida Creek	*R. mucronata*	L	10.13 ± 0.1^ab^	29.07 ± 0.1^a^	10.07 ± 0.1^abcd^
BB3	*Alternaria*	Tudor Creek	*R. mucronata*	B	7.07 ± 0.1^ab^	19.17 ± 0.1^a^	6.07 ± 0.1^cd^
BB6	*Alternaria*	Mida Creek	*R. mucronata*	B	10.03 ± 0.1^ab^	12.07 ± 0.1^ab^	10.13 ± 0.1^abcd^
	Negative control				0.00 ± 0.0^b^	0.00 ± 0.0^b^	0.00 ± 0.0^e^
	Positive control				28.10 ± 0.1	28.30 ± 0.2	31.70 ± 0.1
LC3	*Penicillium*	Mida Creek	*A. marina*	L	0.00 ± 0.0^b^	10.13 ± 0.1^ab^	10.03 ± 0.1^abcd^
LC5	*Penicillium*	Tudor Creek	*A. marina*	L	12.07 ± 0.1^ab^	8.07 ± 0.1^ab^	10.10 ± 0.1^abcd^
RC6	*Penicillium*	Mida Creek	*A. marina*	R	0.00 ± 0.0^b^	8.03 ± 0.1^ab^	0.00 ± 0.0^e^
	Negative control				0.00 ± 0.0^b^	0.00 ± 0.0^b^	0.00 ± 0.0^e^
	Positive control				32.20 ± 0.1	27.09 ± 0.1	37.10 ± 0.1
LB2	*Cladosporium*	Mida Creek	*R. mucronata*	L	10.23 ± 0.1^ab^	8.03 ± 0.1^ab^	12.03 ± 0.1^abc^
LB4	*Cladosporium*	Tudor Creek	*R. mucronata*	L	15.07 ± 0.1^ab^	10.07 ± 0.1^ab^	8.07 ± 0.1^abcde^
RA2	*Cladosporium*	Mida Creek	*B. gymnorrhiza*	R	7.17 ± 0.1^ab^	7.07 ± 0.1^ab^	8.07 ± 0.1^abcde^
	Negative control				0.00 ± 0.0^b^	0.00 ± 0.0^b^	0.00 ± 0.0^e^
	Positive control				35.40 ± 0.1	28.07 ± 0.1	37.40 ± 0.1
RA3	*Aspergillus*	Mida Creek	*B. gymnorrhiza*	R	0.00 ± 0.0^b^	0.00 ± 0.0^b^	0.00 ± 0.0^e^
BA5	*Aspergillus*	Tudor Creek	*B. gymnorrhiza*	B	10.03 ± 0.1^ab^	10.03 ± 0.1^ab^	10.03 ± 0.1^abcd^
BA6	*Aspergillus*	Mida Creek	*B. gymnorrhiza*	B	0.00 ± 0.0^b^	0.00 ± 0.0^b^	0.00 ± 0.0^e^
BB8	*Aspergillus*	Gazi Bay	*R. mucronata*	B	0.00 ± 0.0^b^	10.07 ± 0.1^ab^	19.07 ± 0.1^a^
BC3	*Aspergillus*	Gazi Bay	*A. marina*	B	8.03 ± 0.1^ab^	9.03 ± 0.1^ab^	9.03 ± 0.1^bcd^
BC8	*Aspergillus*	Tudor Creek	*A. marina*	B	9.03 ± 0.1^ab^	8.07 ± 0.1^ab^	9.03 ± 0.1^bcd^
BC9	*Aspergillus*	Mida Creek	*A. marina*	B	0.00 ± 0.0^b^	9.03 ± 0.1^ab^	0.00 ± 0.0^e^
BD4	*Aspergillus*	Gazi Bay	*X. granatum*	B	11.03 ± 0.1^ab^	8.03 ± 0.1^ab^	9.03 ± 0.1^bcd^
BE5	*Aspergillus*	Gazi Bay	*H. littoralis*	B	17.03 ± 0.1^a^	16.03 ± 0.1^a^	21.23 ± 0.3^a^
	Negative control				0.00 ± 0.0^b^	0.00 ± 0.0^b^	0.00 ± 0.0^e^
	Positive control				28.30 ± 0.1	29.30 ± 0.4	29.20 ± 0.3
BA10	*Fusarium*	Gazi Bay	*B. gymnorrhiza*	B	8.03 ± 0.1^ab^	8.03 ± 0.1^ab^	9.03 ± 0.1^bcd^
BC6	*Fusarium*	Mida Creek	*A. marina*	B	7.17 ± 0.1^ab^	10.07 ± 0.1^ab^	12.07 ± 0.1^abc^
	Negative control				0.00 ± 0.0^b^	0.00 ± 0.0^b^	0.00 ± 0.0^e^
	Positive control				21.03 ± 0.1	24.10 ± 0.1	28.60 ± 0.1
RB4	*Lasiodiplodia*	Mida Creek	*R. mucronata*	R	8.03 ± 0.1^ab^	10.07 ± 0.1^ab^	11.03 ± 0.1^abc^
BB4	*Lasiodiplodia*	Tudor Creek	*R. mucronata*	B	19.03 ± 0.1^ab^	7.07 ± 0.1^ab^	19.03 ± 0.1^a^
BB7	*Lasiodiplodia*	Mida Creek	*R. mucronata*	B	0.00 ± 0.0^b^	9.03 ± 0.1^ab^	18.07 ± 0.1^ab^
	Negative control				0.00 ± 0.0^b^	0.00 ± 0.0^b^	0.00 ± 0.0^e^
	Positive control				25.70 ± 0.1	30.10 ± 0.1	39.70 ± 0.1
BA8	*Nigrospora*	Mida Creek	*B. gymnorrhiza*	B	10.07 ± 0.1^ab^	12.07 ± 0.1^ab^	7.07 ± 0.1^bcde^
BA11	*Nigrospora*	Tudor Creek	*B. gymnorrhiza*	B	15.07 ± 0.1^ab^	26.07 ± 0.1^a^	20.03 ± 0.1^a^
BC7	*Nigrospora*	Mida Creek	*A. marina*	B	11.03 ± 0.1^ab^	10.07 ± 0.1^ab^	10.07 ± 0.1^abcd^
RC3	*Nigrospora*	Mida Creek	*A. marina*	R	0.00 ± 0.0^b^	16.03 ± 0.1^a^	10.07 ± 0.1^abcd^
	Negative control				0.00 ± 0.0^b^	0.00 ± 0.0^b^	0.00 ± 0.0^e^
	Positive control				34.60 ± 0.1	27.00 ± 0.1	30.10 ± 0.1
BC2	*Chaetomium*	Gazi Bay	*A. marina*	B	9.03 ± 0.1^ab^	11.03 ± 0.1^ab^	10.03 ± 0.1^abcd^
	Negative control				0.00 ± 0.0^b^	0.00 ± 0.0^b^	0.00 ± 0.0^e^
	Positive control				25.03 ± 0.1	25.03 ± 0.1	28.07 ± 0.1

Isolates are coded depending on the part of the tree sampled; prefix letters L, B, and R represent leaf, branch, and root tissue of the mangrove species, *respectively*, followed by letters A, B, C, D, and E representing *B. gymnorrhiza, R. mucronata, A. marina, X. granatum,* and *H. littoralis mangrove species, respectively, and then a number of the sample collected from the* mangrove species is isolated. Means followed by the same superscript letter(s) in each group are not significantly different at *α* = 0.05 (Fisher LSD, 95% confidence). The significance of each group is represented by superscript letters (a, b, and c). The distinct letters indicate statistically significant differences between the groups. Therefore, when two groups are assigned different superscript letters, their results are statistically different, and any observed difference between them is considered significant.

**Table 4:** Antibacterial activities of ethyl acetate and butanol extracts of fungal mycelia.

Endophytic fungi	Ethyl acetate and butanol extracts' inhibition zone diameters (mm)					
	Gram-negative bacteria				Gram-positive bacteria	
	*Escherichia coli (ATCC 25922*)		*Pseudomonas aeruginosa* (*ATCC 27853*)		*Staphylococcus aureus* (ATCC 25923)	
	Extracts					
Sample ID	Ethyl acetate	Butanol	Ethyl acetate	Butanol	Ethyl acetate	Butanol
Negative control	0.00 ± 0.0	0.00 ± 0.0	0.00 ± 0.0	0.00 ± 0.0	0.00 ± 0.0	0.00 ± 0.0
M1	10.07 ± 0.1^a^	10.07 ± 0.1^b^	11.03 ± 0.1^a^	10.07 ± 0.1^a^	15.07 ± 0.1^a^	14.07 ± 0.1^a^
M2	12.07 ± 0.1^a^	10.07 ± 0.1^b^	15.07 ± 0.1^a^	15.07 ± 0.1^a^	18.07 ± 0.1^a^	16.03 ± 0.1^a^
M3	12.07 ± 0.1^a^	10.07 ± 0.1^b^	9.03 ± 0.1^a^	11.03 ± 0.1^a^	15.07 ± 0.1^a^	15.07 ± 0.1^a^
M4	9.03 ± 0.1^a^	10.07 ± 0.1^b^	12.07 ± 0.1^a^	12.07 ± 0.1^a^	15.07 ± 0.1^a^	12.07 ± 0.1^a^
M5	16.03 ± 0.1^a^	11.03 ± 0.1^b^	11.03 ± 0.1^a^	8.03 ± 0.1^a^	11.03 ± 0.1^a^	9.03 ± 0.1^a^
M6	10.07 ± 0.1^a^	14.07 ± 0.1^a^	9.03 ± 0.1^a^	10.07 ± 0.1^a^	10.07 ± 0.1^a^	13.07 ± 0.1^a^
M7	0.00 ± 0.0^a^	9.03 ± 0.1^b^	9.03 ± 0.1^a^	10.07 ± 0.1^a^	10.07 ± 0.1^a^	9.03 ± 0.1^a^
M8	12.07 ± 0.1^a^	16.03 ± 0.1^a^	16.03 ± 0.1^a^	10.07 ± 0.1^a^	15.07 ± 0.1^a^	20.03 ± 0.1^a^
Positive control	28.06 ± 3.4	28.06 ± 3.4	29.15 ± 3.9	29.15 ± 3.9	33.40 ± 2.3	33.40 ± 2.3

Mycelia isolates denoted by genera as M1, *Alternaria*; M2, *Penicillium*; M3, *Cladosporium*; M4, *Aspergillus*; M5, *Fusarium*; M6, *Lasiodiplodia*; M7, *Nigrospora*; and M8, *Chaetomium*. ^∗^Concentration of positive control (Streptomycin) 20 mg/ml in distilled water. Means followed by the same superscript letter in each group are not significantly different at *α* = 0.05 (Fisher LSD, 95% confidence). The significance of each group is represented by superscript letters (a, b, and c). The distinct letters indicate statistically significant differences between the groups. Therefore, when two groups are assigned different superscript letters, it indicates that their results are statistically different, and any observed difference between them is considered to be significant.

**Table 5:** Minimum inhibitory concentrations (MIC) of ethyl acetate and butanol extracts of the fungal isolates against the tested human pathogenic bacteria.

MIC (mg/ml)				
Sample ID	Extract	*Escherichia coli*	*Pseudomonas aeruginosa*	*Staphylococcus aureus*
BD4	Ethyl acetate	3.88 ± 0.01^b^	4.96 ± 0.01^b^	4.90 ± 0.00^b^
BA11		5.32 ± 0.01^a^	4.62 ± 0.01^b^	3.05 ± 0.01^c^
	Butanol	—	4.96 ± 0.01^b^	—
BE5	Ethyl acetate	2.86 ± 0.01^d^	5.17 ± 0.01^a^	2.96 ± 0.01^d^
LB4		4.94 ± 0.01^a^	3.23 ± 0.01^c^	3.69 ± 0.01^c^
RC6	Ethyl acetate	—	3.31 ± 0.01^c^	—
RC3		—	4.58 ± 0.01^b^	—
LB1	Butanol	—	4.57 ± 0.01^b^	—
Control (streptomycin)		3.77 ± 0.00^b^	3.84 ± 0.00^c^	4.5 ± 0.00^b^

Isolates denoted as LB4 and LB1, leaf isolates of *R. mucronata*; RC6 and RC3, root isolates of *A. marina*; and BD4, BA11 and BE5, branch isolates of *X. granatum*, *B. gymnorrhiza* and *H. littoralis* species; ‘—' denotes values not determined. Data presented as means ± SD of three replicates. ^∗^Concentration of positive control (Streptomycin) 20 mg/ml in distilled water. Means followed by the same superscript letter in each group are not significantly different at *α* = 0.05 (Fisher LSD, 95% confidence). The significance of each group is represented by superscript letters (a, b, and c). The distinct letters indicate statistically significant differences between the groups. Therefore, when two groups are assigned different superscript letters, it indicates that their results are statistically different, and any observed difference between them is considered to be significant.

## Data Availability

The data presented in this study are available upon request from the corresponding authors.
